# Supplementary material for: Intensity adaptive optics
Source: Light Sci Appl. 2025 Mar 19;14:128. doi: 10.1038/s41377-025-01779-0 (PMC11923252; doi:10.1038/s41377-025-01779-0)
Supplement: Supplementary file 1 — SI [file 41377_2025_1779_MOESM1_ESM.pdf]

Supplementary Information

Intensity adaptive optics

Zimo Zhao[1,†], Yifei Ma[1,†], Zipei Song[1,†], Jacopo Antonello[1], Jiahe Cui[1], Binguo Chen[2], Jingyu Wang[1], Bangshan Sun[1], Honghui He[2], Lin Luo[3], Julian A.J. Fells[1], Steve J. Elston[1], Martin J. Booth[1], Stephen M. Morris[1], and Chao He[1,*]

[1]*Department of Engineering Science, University of Oxford, Parks Road, Oxford, OX1 3PJ, UK*
[2]*Guangdong Engineering Center of Polarization Imaging and Sensing Technology, Tsinghua Shenzhen International Graduate School, Tsinghua University, Shenzhen 518055, China*
[3]*College of Engineering, Peking University, Beijing 100871, China*
[†]*These authors contributed equally to this work*
[*]*Corresponding author:* chao.he@eng.ox.ac.uk

Supplementary Note 1:

Experiment configuration for intensity error correction

The configuration of the correction system employed in this work is presented in Fig. S1. A He-Ne laser beam (Melles Griot, 05-LHP-171) emitting at a wavelength of 632.8 nm with an attenuation filter (AF) in front was expanded and directed towards the spatial light modulator (SLM) (Hamamatsu, X10468-01) and a deformable mirror (DM) (Boston Micromachines Corporation, Multi-3.5). The SLM was used in combination with the DM functioning as the intensity adaptive optics (I-AO) corrector. The SLM was positioned between a pair of crossed polarisers (P1 and P2), with the fast axis of the nematic liquid crystal (NLC) in each SLM pixel aligned at 45° to the transmission axes of the polarisers. The pupil plane from the surface of the SLM was relayed to the surface of the DM, and then further relayed to the designed pupil plane. Various intensity errors can be used in this configuration, such as a controllable error, a biomedical sample or a material sample. Note that all intensity manipulation occurs at the pupil plane, while data are recorded in the far-field. In this work, we mainly use the controllable intensity error introduced by an SLM sandwiched with a pair of crossed polarisers. This SLM block allows for precise manipulation of intensity error in a pixelated manner for testing and verification of the proposed methods.

Depending on the correction methods used (i.e., sensorless or sensor-based), different elements were incorporated into the system via flip mirror FM1 to facilitate the correction process. In the sensor-based method, the pupil plane was directly imaged onto the CCD, allowing precise measurement of intensity errors. The compensatory intensity was calculated by comparing the measured pupil profile with a uniform intensity distribution. In the sensorless method, the pupil plane was focused through a lens, and image metrics were applied to estimate the compensatory intensity based on the deviations from the ideal focal distribution, without a direct imaging of the pupil plane.

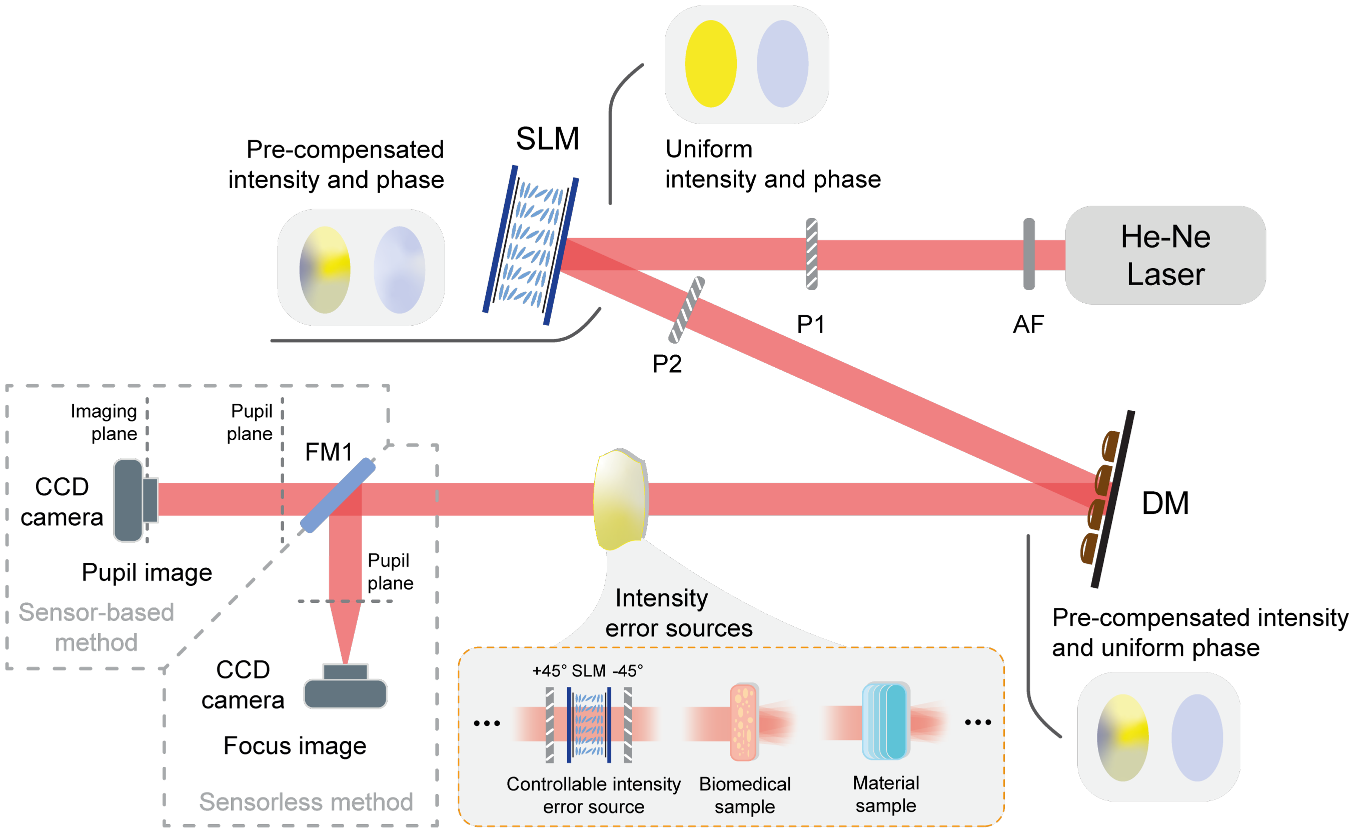

Figure S1: Configuration of the experiment system for I-AO. A He-Ne laser emitting at 632.8 nm with an attenuation filter (AF) serves as the illumination source. Lenses are omitted in the illustration for clarity and simplicity. The beam is expanded and passes through an SLM paired with a DM. The SLM incorporates crossed polarisers (P1 and P2) aligned at +45° and −45° relative to the SLM director. A 4f system relays the optical path from the SLM to the DM, and then further to the final pupil plane and its conjugate planes. Various intensity error sources can be used in this configuration, such as a SLM based controllable error source, a biomedical sample or a material sample. The system supports both sensor-based and sensorless correction methods by adjusting the flip mirror (FM1). In the sensor-based method, pupil planes are directly imaged onto a CCD, whereas the sensorless method evaluates the focus of the beam at the focal plane to estimate the error without direct imaging.

Supplementary Note 2:

Dual-loop correction approach

In conventional phase adaptive optics (AO), correction typically relies on a single feedback loop, either by directly measuring the wavefront at the pupil plane (sensor-based) or inferring phase aberrations from focus intensity (sensorless). However, the single-loop approach is insufficient for compensating intensity errors. To overcome this limitation, we propose a dual-loop correction mechanism within the I-AO system. Loop 1 focuses on restoring intensity uniformity at the pupil plane, which is the primary objective of the whole mechanism. Loop 2 complements this by adjusting the intensity attenuator at the illumination source to recover total energy, while preserving the uniformity achieved in the first loop. This method structures intensity correction into two prioritised tasks: restoring uniformity and recovering total intensity. Fig. S2 illustrates this dual-loop approach, applicable to both sensor-based and sensorless mechanisms.

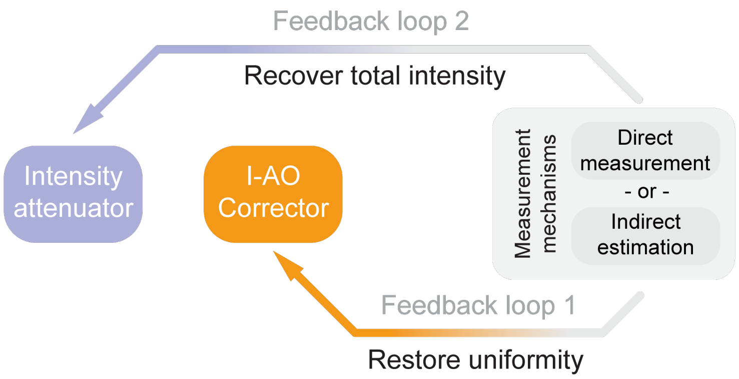

Figure S2: Illustration of the dual-loop correction approach in the I-AO system. This dual-loop method addresses the effect of intensity errors in two stages. These feedback loops, utilised in both sensor-based and sensorless mechanisms, enable effective I-AO correction procedures.

Supplementary Note 3:

Spatial light modulator (SLM) calibration for I-AO corrector

An SLM is positioned within the I-AO corrector between two orthogonal polarisers. Both polarisers are set at a 45° angle relative to the director of NLC in the SLM, enabling pixelated intensity modulation through the NLC's Fréedericksz transition. The transmittance (T) of light through the NLC layer is given by:

$$T = \sin^2(2\chi)\sin^2\left(\frac{\pi\Delta n d}{\lambda}\right) \quad (1)$$

where χ represents the 45° angle between the polariser and the NLC director, Δn denotes the NLC's birefringence, d is the distance that light travels within the NLC layer, and λ is the wavelength.

Intensity modulation in the I-AO corrector is achieved by adjusting the voltage applied to the NLC within the SLM to change its birefringence. An 8-bit grayscale image with the same resolution as the SLM panel is converted into voltage values based on a manufacturer-defined grayscale-to-voltage map. However, due to surface unevenness in the SLM, the NLC layer thickness varies across the panel, affecting modulation accuracy. A pixel-specific look-up table (LUT) is created to match pixel values with their modulated intensities, allowing precise and rapid intensity adjustments by quickly identifying the correct pattern to load onto the SLM during the correction process. The LUT for each SLM pixel is derived by mapping intensity modulation to corresponding grayscale values. To maximise its dynamic range, the laser intensity is adjusted using a neutral density (ND) filter, and the exposure time of a monochrome camera is also optimised. These parameters are then held constant throughout the calibration routine.

To efficiently capture intensity modulation responses across all SLM pixels, uniform grayscale images (termed flat value F_i) are applied to the SLM. With an 8-bit pixel modulation depth, integer flat values $F_i = i$, where $i \in [0,255]$, are tested for each SLM pixel. Each integer flat value F_i is applied to all N pixels of the SLM and incremented from 0 to 255, resulting in 256 captured images of the full active region of the SLM. Interpolation is used to address the resolution difference between the SLM and the camera sensor, ensuring an accurate one-to-one mapping between each SLM pixel and each camera pixel, thus eliminating potential issues related to resolution mismatch. For each SLM pixel n, an intensity response P_{n_i} is recorded for every flat value F_i, generating a modulation range $[P_{n_0}, P_{n_1}, \cdots, P_{n_{255}}]$. This process captures the modulation profile of the entire SLM panel and enables precise calibration of pixel-specific intensity responses, often represented by trigonometric functions within the modulation range[1].

To ensure the mapping is unique between SLM pixel values and intensity modulation, flat values F_{S_n} and F_{L_n}, corresponding to minimum intensity $P_{n_{S_n}}$ and maximum intensity $P_{n_{L_n}}$, are identified for each SLM pixel n. Intensity values within the range $[P_{n_{S_n}}, P_{n_{L_n}}]$ are used for I-AO compensation, while values outside this range are discarded. Thus, for each SLM pixel n, setting the pixel value between F_{S_n} and F_{L_n} yields a unique mapping to the intensity range of $[P_{n_{S_n}}, P_{n_{L_n}}]$ on the monochromatic camera. This approach establishes the relationship between each SLM pixel's flat value and its modulated intensity range, which is then encoded into the LUT along with the valid pixel value range. This calibration procedure is illustrated in the flowchart in Fig. S3.

To achieve a desired output intensity using the I-AO corrector, each SLM pixel is modulated by selecting a flat value F_i within $[F_{S_n}, F_{L_n}]$ that produces an intensity P_{n_i} closely matching the target P_{n_T} on the monochrome camera, as determined by the pre-calibrated LUT. Subsequently, conventional phase AO correction is applied using a deformable mirror (DM) to correct for phase errors induced by the SLM and other optical elements. For a uniform output intensity, a single target intensity P_{n_T} is set across the monochrome camera, validating the unique SLM pattern that achieves uniform intensity at the pupil plane. In this configuration, the focal plane image (**Figure 2**(b) in the Main article) exhibits an ideal focal intensity distribution resembling a standard Airy disk shape.

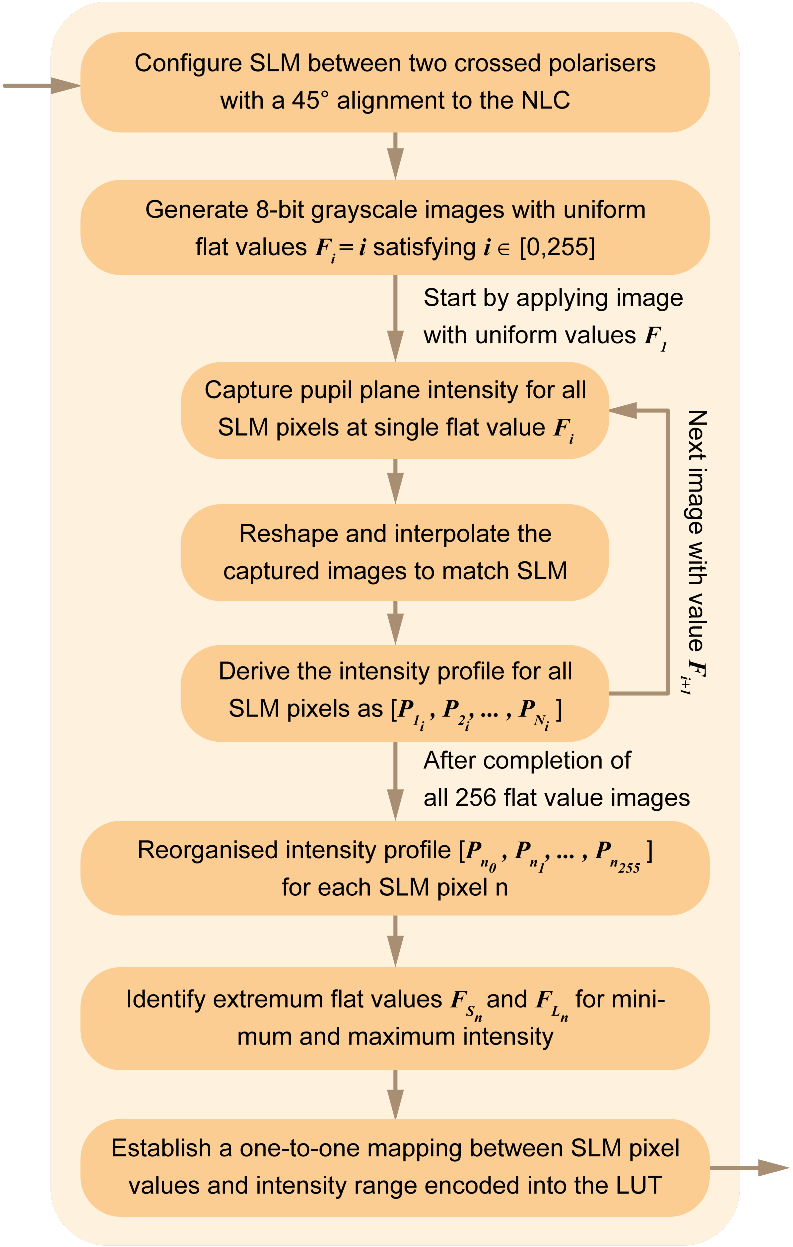

Figure S3: Flowchart detailing the calibration process of the SLM within the I-AO corrector. The diagram outlines the configuration of the SLM with crossed polarisers, the systematic measurement of intensity modulation across flat values, and the development of a pixel-specific LUT to enable precise intensity correction.

Supplementary Note 4:

Experimental procedures for sensor-based I-AO

The sensor-based I-AO approach directly measures intensity distribution at the pupil plane using a sensor. Initially, the SLM is set to a flat state (see **Supplementary Note 3**), and pupil plane images are captured as the reference for intensity errors correction, with focal plane images used solely for validation. External intensity errors are then introduced into the system, and the distorted intensity profile is captured by the intensity sensor, initiating a dual-feedback loop for sensor-based correction.

In Loop 1, the intensity uniformity is restored by calculating the intensity loss ΔP_n for each pixel n, determined by comparing captured intensity values P_{n_C} with the target level P_{n_T}, where $\Delta P_n = P_{n_T} - P_{n_C}$. A new value $\tilde{P}_n = P_{n_T} + \Delta P_n$ is set on the monochrome camera to compensate for the intensity errors, resulting in an updated SLM pattern based on the pre-calibrated LUT. The corrected pupil intensity is then assessed to confirm optimal beam uniformity restoration. Subsequently, conventional phase AO correction is applied to address phase errors introduced by the SLM during intensity correction. Then, if we examine the focal plane, the focus intensity distribution (FID) will resemble an ideal Airy disk, confirming uniform intensity at the pupil plane.

After rectifying the intensity profile uniformity in Loop 1, Loop 2 aims to recover the total intensity by adjusting the attenuation filter at the illumination source, guided by feedback from the camera-measured intensity level. A flowchart in Fig. S4 summarises this sensor-based correction methodology.

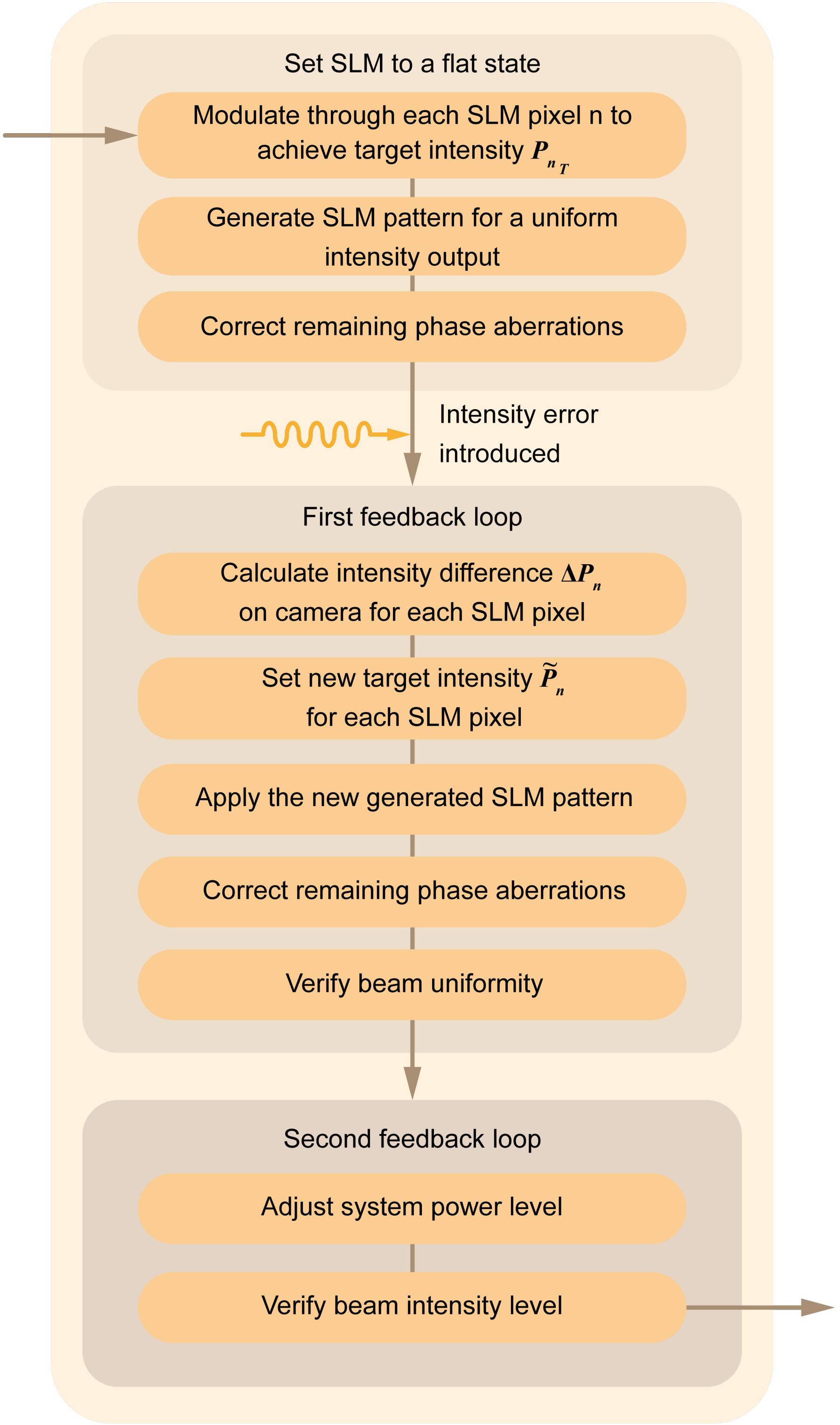

Figure S4: Flowchart illustrating the sensor-based correction methodology in the I-AO system. Key steps include initial SLM flat-state setup, beam uniformity restoration through the first feedback loop, and total intensity recovery via the second feedback loop.

Supplementary Note 5:

Necessity of intensity correction in the pupil plane

In the context of optical wave propagation, the wave in the pupil plane of a lens is expressed as

$$U(x, y) = A(x, y)P(x, y)e^{i\phi(x,y)} \tag{2}$$

where $A(x, y)$ represents the amplitude at the pupil, $P(x, y)$ represents the pupil function, which is unity inside the pupil and zero outside, and $\phi(x, y)$ is the corresponding phase of the wave. As the wave propagates to the focal plane (far field) through the lens, the complex amplitude becomes:

$$U_f(u, v) = \frac{e^{-i\frac{\pi}{\lambda f}(u^2+v^2)}}{i\lambda f} \cdot \mathcal{F}\{A(x, y)P(x, y)e^{i\phi(x,y)}\} \tag{3}$$

where u, v are coordinates in the focal plane, f is the focal length, λ is the wavelength and $\mathcal{F}$ denotes the Fourier transform. Therefore, the intensity in the focal plane is given by:

$$I_f(u, v) = |U_f(u, v)|^2 = \frac{1}{(\lambda f)^2}\left|\mathcal{F}\{A(x, y)P(x, y)e^{i\phi(x,y)}\}\right|^2 \tag{4}$$

And the Fourier transform of the initial wave $U(x, y)$ in the pupil plane is expressed as:

$$\mathcal{F}\{A(x, y)P(x, y)e^{i\phi(x,y)}\}(u, v) = \int_{-\infty}^{+\infty}\int_{-\infty}^{+\infty} A(x, y)\, P(x, y)e^{i\phi(x,y)}e^{-i2\pi(ux+vy)}dxdy \tag{5}$$

In conventional phase-only AO, the primary focus is on correcting phase aberrations to improve the far-field intensity pattern. For instance, when the amplitude is uniform ($A(x, y) = A_0$), the initial field at the pupil plane can be simplified into $U(x, y) = A_0P(x, y)e^{i\phi(x,y)}$ and the corresponding Fourier transform after the lens gives:

$$\mathcal{F}\{U(x, y)\}|_{A(x,y)=A_0} = A_0\int_{-\infty}^{+\infty}\int_{-\infty}^{+\infty} P(x, y)e^{i\phi(x,y)}e^{-i2\pi(ux+vy)}dxdy \tag{6}$$

If the phase profile $\phi(x, y)$ is non-uniform, the constructive and destructive interference leads to spatially varying intensity in the focal plane. Only a constant or linearly varying phase profile ($\phi(x, y) = \phi_0$ or $\phi(x, y) = ax + by$) leads to a far-field intensity forming an ideal Airy disk with the same original intensity level, which corresponds to the Fourier transform of the pupil function $P(x, y)$. By modifying the phase into other profiles, conventional AO can achieve the intended far-field intensity distribution but cannot preserve both the ideal Airy disk shape and the original intensity level.

However, the situation becomes complex when the amplitude $A(x, y)$ in the pupil plane is non-uniform. The intensity profile at the pupil plane $I(x, y) = |U(x, y)|^2 = A(x, y)^2$ depends directly on the initial amplitude $A(x, y)$, while the phase $\phi(x, y)$ only governs wave propagation after the pupil rather than intensity directly. Ideally, the final Fourier transform should give the shape of an ideal Airy disk after the correction. Thus, the following expression should hold:

$$\int_{-\infty}^{+\infty}\int_{-\infty}^{+\infty} A(x, y)P(x, y)e^{i\phi(x,y)}e^{-i2\pi(ux+vy)}dxdy = \int_{-\infty}^{+\infty}\int_{-\infty}^{+\infty} cP(x, y)e^{-i2\pi((u-\frac{a}{2\pi})x+(v-\frac{b}{2\pi})y)} \tag{7}$$

where c is a scalar constant, $a/2\pi$ and $b/2\pi$ control the translational position of $\mathcal{F}\{P(x, y)\}$ in the focal plane, and u, v are coordinates in the focal plane. Therefore,

$$A(x, y)P(x, y)e^{i\phi(x,y)} = cP(x, y)e^{i(ax+by)} \tag{8}$$

$P(x, y)$ is the common pupil function which can be eliminated, and the following equations can be derived by equating the real and imaginary parts to obtain an identical transformation of the pupil function $P(x, y)$:

$$\begin{cases} A(x, y) \cdot \cos\phi(x, y) = c \cdot \cos(ax + by) \\ A(x, y) \cdot \sin\phi(x, y) = c \cdot \sin(ax + by) \end{cases} \tag{9}$$

Eq. 9 holds for $\forall x, y \in \mathbb{R}$. Therefore, to obtain the ideal Airy disk shape with original intensity level, $A(x,y)$ and $\phi(x,y)$ should satisfy:

$$\begin{cases} A(x,y) = c \\ \phi(x,y) = (a + 2m\pi)x + (b + 2n\pi)y, \;\; m,n \in \mathbb{Z} \end{cases} \tag{10}$$

So, if conventional phase-only AO is applied to correct the wavefront, no satisfactory $\phi(x,y)$ can be achieved as long as the amplitude $A(x,y)$ in the pupil plane remains non-uniform (where $A(x,y) \neq c$). Moreover, any high-order phase profile frequently used in conventional phase AO will prevent the formation of an ideal Airy disk. This highlights the necessity of having the ability to control the intensity profile directly at the pupil plane. It also explains why phase-only AO cannot correct intensity errors originating at the pupil plane, as demonstrated by the experimental results in Fig. 2(a).

Moreover, for the ideal case where the phase is uniform ($\phi(x,y) = \phi_0$), the non-uniform amplitude profile $A(x,y)$ convolves with the pupil function $P(x,y)$ and alter the result of its Fourier transform, causing intensity errors in the focal plane. This relationship is described by the Fourier transform after the lens,

$$\mathcal{F}\{U(x,y)\}|_{A(x,y)=A_0} = e^{i\phi_0} \int_{-\infty}^{+\infty} \int_{-\infty}^{+\infty} A(x,y) P(x,y) e^{-i2\pi(ux+vy)} dxdy \tag{11}$$

Only when the amplitude profile $A(x,y)$ is uniform ($A(x,y) = A_0$) does the focal plane result become the ideal Airy disk shape with original intensity level.

This highlights the importance of achieving intensity uniformity at the pupil plane using I-AO, as demonstrated by the experimental results in Fig. 2(b). The I-AO system detailed in **Supplementary Notes 1**, which employs an SLM positioned between two polarisers and cascaded with a DM, enables direct control of the intensity profile $A(x,y)$ at the pupil plane. This novel approach introduces a new degree of freedom to the AO toolkit by restoring intensity uniformity without altering the phase profile $\phi(x,y)$, and effectively addresses the limitations of conventional phase-only AO methods in handling the amplitude non-uniformity.

Supplementary Note 6:

Intensity Zernike Modes

Our proposed sensorless I-AO method infers and corrects intensity errors by using the FID profile at the focal plane as feedback for the corrector, applying a correction mechanism like that of conventional sensorless phase AO. However, instead of conventional phase AO Zernike modes, we introduce a new set of intensity-based Zernike modes specifically designed for estimating the intensity errors – as intensity correction inherently requires non-negative modes. For simplicity and to facilitate a proof-of-concept demonstration, we use Zernike polynomials as the basis for constructing intensity correction modes; however, other mode formats may be explored and constructed for further optimisations, as discussed in the main article.

These modified Zernike polynomials differ in both the range of possible values and application strategy and can be expressed as:

$$\mathcal{Z}_n^m(\rho,\theta) = \frac{1}{2}\left[c_n^m R_n^{|m|}(\rho)\Theta_n^m(\theta)\right] + \frac{1}{2} \tag{12}$$

where:

$$c_n^m = 1 \tag{13}$$

$$\Theta_n^m(\theta) = \begin{cases} \cos(m\theta) & m \geq 0 \\ -\sin(m\theta) & m < 0 \end{cases} \tag{14}$$

$$R_n^{|m|}(\rho) = \sum_{l=0}^{(n-|m|)/2} \frac{(-1)^l (n-l)!}{l!\left[\frac{1}{2}(n+|m|-l)\right]!\left[\frac{1}{2}(n-|m|-l)\right]!}\rho^{n-2l} \tag{15}$$

m and n denote the azimuthal frequency and radial degree, respectively[2]. The total number of intensity Zernike modes used in the I-AO corrector is typically determined by the characteristics of the measured sample. For demonstration proposes, we use the first 15 intensity Zernike modes in this work for sensorless I-AO correction, with additional modes available for correcting more complex errors.

Because of the geometrical crosstalk between intensity and phase during SLM modulation, additional phase errors are introduced. The state of polarisation (SOP) and scalar phase across the SLM can be modelled using Jones calculus to represent the light passing through[3-5].

Note that in this work, our primary focus is on intensity correction. Therefore, we compensate the residual phase errors to minimise their influence on the FID. However, we acknowledge that in the future, phase modulation may be harnessed to construct more effective correction modes.

For each point on the pupil plane, the change in SOP and phase can be modelled as:

$$\boldsymbol{j_2} = \mathrm{J} \cdot \boldsymbol{j_1} \tag{16}$$

where $\boldsymbol{j_1}$ and $\boldsymbol{j_2}$ are the Jones vectors before and after the change in SOP and scalar phase, respectively, and J is a 2 × 2 Jones matrix that models the optical effect of an SLM positioned between a pair of crossed polarisers. In this paper, we focus exclusively on retardance and phase modulation for fully polarised light. As a result, J can be expressed as

$$\mathrm{J} = e^{i\phi} \cdot U \tag{17}$$

where ϕ is the scalar phase applied to the SLM and U is a special unitary matrix (SU) describing the change in SOP. This latter matrix can also be parametrised as[6]

$$U = SU2(\boldsymbol{Q}, \phi) = \cos\left(\frac{\phi}{2}\right)\mathbf{I} + \sin\left(\frac{\phi}{2}\right) \cdot (n_1\sigma_1 + n_2\sigma_2 + n_3\sigma_3) \tag{18}$$

where $\mathbf{I}$ is the 2×2 identity matrix, and σ_1, σ_2, σ_3 are the Pauli matrices defined as

$$\sigma_1 = \begin{bmatrix} 1 & 0 \\ 0 & -1 \end{bmatrix}, \sigma_2 = \begin{bmatrix} 0 & 1 \\ 1 & 0 \end{bmatrix}, \sigma_3 = \begin{bmatrix} 0 & i \\ -i & 0 \end{bmatrix} \tag{19}$$

Note that, σ_3 is defined differently than in some other sources[3-5,7,8]. Here, the coefficients n_1, n_2, n_3 belong to a vector $\boldsymbol{Q} = [n_1, n_2, n_3]$ with unit norm, i.e., $\|\boldsymbol{Q}\| = 1$. The SLM, which is based on the NLC, exhibits a phase delay between its two optical axes. This delay is governed by its birefringence Δn, which can be modulated by an external electric field. In the SLM, the phase delay introduced by each pixel is determined by the pixel value applied and is defined as:

$$\phi = \frac{2\pi \cdot \Delta n \cdot d}{\lambda} = 2\mathrm{arcsin}\left(\sqrt{\frac{T}{\sin^2 2\chi}}\right) \tag{20}$$

where the definition of T, χ, Δn, d and λ follow the definition as described in **Supplementary Note 3** and the phase delay ϕ is controlled by the pixel values. The effect of a single pixel on the SLM can then be expressed by

$$\mathrm{J}_{LC} = \begin{bmatrix} e^{i\phi} & 0 \\ 0 & 1 \end{bmatrix} = e^{i\frac{\phi}{2}} \begin{bmatrix} e^{i\frac{\phi}{2}} & 0 \\ 0 & e^{-i\frac{\phi}{2}} \end{bmatrix} = e^{i\frac{\phi}{2}} \cdot SU2(\mathbf{H}, \phi) \tag{21}$$

where $\mathbf{H} = [1; 0; 0]$. In our experiment, the SLM is configured with a reflective backplane behind the pixel array. Consequently, the Jones vector after reflection from the SLM can be expressed as:

$$\mathrm{J}_{SLM} = \mathrm{J}_r \cdot e^{i\frac{\phi}{2}} \cdot SU2(\mathbf{H}, \phi) \tag{22}$$

where $J_r = \sigma_1 = iSU2(\mathbf{H}, \pi)$ represents the reflection from the backplane of the SLM[9]. The Jones matrices for linear polarisers at $+45°$ and $-45°$ are given, respectively, by

$$\mathrm{J}_{+45} = \frac{1}{2}\begin{bmatrix} 1 & 1 \\ 1 & 1 \end{bmatrix} \tag{23}$$

$$\mathrm{J}_{-45} = \frac{1}{2}\begin{bmatrix} 1 & -1 \\ -1 & 1 \end{bmatrix} \tag{24}$$

Therefore, the Jones matrix of each pixel across the pupil of the SLM module is represented as:

$$\mathrm{J} = \mathrm{J}_{-45} \cdot \mathrm{J}_{SLM} \cdot \mathrm{J}_{+45} \tag{25}$$

This relationship leads to the input and output Jones vectors before and after SLM modulation:

$$\boldsymbol{j_2} = \mathrm{J}_{-45} \cdot \mathrm{J}_{SLM} \cdot \mathrm{J}_{+45} \cdot \boldsymbol{j_1} = e^{i\frac{\phi+\pi}{2}} \cdot \mathrm{J}_{+45} \cdot SU2(\mathbf{H}, \phi) SU2(\mathbf{H}, \pi) \cdot \mathrm{J}_{-45} \cdot \boldsymbol{j_1} \tag{26}$$

Subsequently, the phase difference between $\boldsymbol{j_1}$ and $\boldsymbol{j_2}$ is computed using the Pancharatnam connection[8,10], which is defined as

$$\Pi(\boldsymbol{j_1}, \boldsymbol{j_2}) = \arg(\boldsymbol{j_1^\dagger j_2}) \tag{27}$$

The DM then compensates for this additional phase by applying $\varphi = -\Pi(\boldsymbol{j_1}, \boldsymbol{j_2})$. Based on the intensity Zernike mode $Z_n^m(\rho, \theta)$ applied to the SLM, the corresponding phase φ can be derived from Eq. 26 and Eq. 27 ranging from 0.5π to π. Thus, each intensity Zernike mode applied onto the SLM will have a companion DM phase pattern to ensure that the I-AO corrector provides pure intensity modulation. The combined use of an SLM with crossed polarisers and a DM forms the foundation of this sensorless I-AO algorithm. The first 15 modified intensity Zernike modes are demonstrated alongside their corresponding phase pattern pairs in Fig. S5.

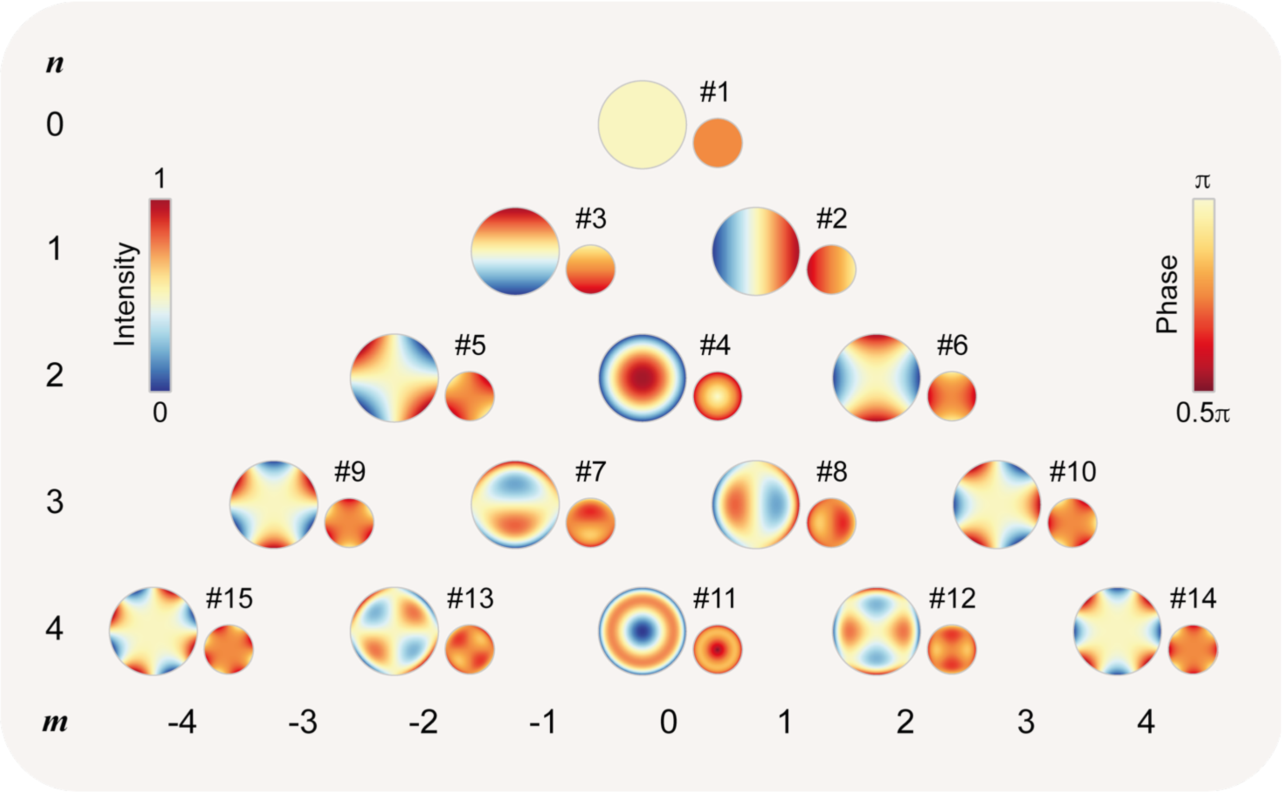

Figure S5: Intensity Zernike modes and their corresponding phase pattern pairs for sensorless I-AO correction. Here, m and n denote the azimuthal frequency and radial degree, respectively. Intensity Zernike modes range between $[0, 1]$, and the additional phases introduced by the SLM range between $[0.5\pi, \pi]$, as shown by the colour bar on the right. The indexing of intensity Zernike modes adheres to Noll's convention.

Supplementary Note 7:

Experimental procedures for sensorless I-AO

Our sensorless I-AO method iteratively applies predefined intensity correction patterns to the I-AO corrector, using focal plane FID profiles to indirectly estimate errors. Inspired by wavefront sensorless algorithms for phase AO, this approach employs a novel set of intensity Zernike modes (detailed in **Supplementary Note 6**) instead of traditional phase modes. Unlike phase AO, where multiple modes can be superimposed for optimisation, sensorless I-AO is constrained by the energy related nature of intensity, which prevents the direct application of orthogonal correction modes used in sensorless phase AO. Therefore, in each iteration, a single intensity Zernike mode with its optimal coefficient is selected to correct the errors.

After SLM calibration (see **Supplementary Note 3)**, the SLM is initially configured to produce a uniform intensity profile. Next, an arbitrary intensity error is introduced, distorting the FID. In the first correction loop, 15 intensity Zernike modes are evaluated to identify the optimal mode and coefficient for restoring the beam uniformity. Unlike traditional Zernike modes used in phase correction, these intensity Zernike modes introduced in **Supplementary Note 6** do not support conventional superposition.

Our method performs a coarse scan through the coefficient range $[0,1]$ for each mode with a step size of 0.2, capturing an FID profile for each step and resetting the I-AO corrector to a flat state before examining the subsequent mode. In contrast, conventional sensorless phase AO algorithms retain previous optimal coefficients and modes, while our approach releases the previous correction mode before applying the next, thereby avoiding intensity saturation at the pupil plane.

To select the optimal mode and coefficient for error correction, FID profile images from the coarse scan are analysed based on focal spot quality. The goal is to identify the intensity Zernike mode and coefficient that restores the focal spot to an ideal Airy disk shape. Selection relies on two criteria: low spatial frequency content in the FID profile images[11] and focal spot circularity. The low spatial frequency criterion helps identify the coefficient of a mode that most closely matches the ideal intensity profile, while circularity assesses whether the selected mode and coefficient restore the desired shape. Circularity C is computed as

$$C = \frac{4A\pi}{P^2} \tag{28}$$

where A is the spot area and P is the perimeter of the target spot. A circularity value of 1 indicates a perfect circle, with larger deviations reflecting a poorer FID profile. During experiments, the optimal coefficient is first determined for each mode using the low spatial frequency algorithm. Circularity C is then calculated for each instance, and the mode yielding a circularity value closest to 1 is selected. For simplicity, among modes with a circularity value close to 1, the mode with the lowest index number is chosen. In this proof-of-concept demonstration, only the best mode is selected for correction; however, this method can sequentially correct multiple modes as long as intensity saturation is avoided.

After identifying the optimal mode for error correction, its coefficient is rescanned over the range $[0,1]$ with a fine increment of 0.05, capturing FID profile images for each coefficient. These images are analysed for the low spatial frequency content to determine the optimal coefficient. The selected mode and optimal coefficient are then applied, and an image is captured at the focal plane for validation. Any residual discrepancy between the actual and ideal focal spot profile is attributed to phase errors introduced by the intensity correction pattern on the SLM. Conventional sensorless phase AO is then applied to address these residual phase errors, further refining the FID profile.

Following the initial correction loop that restores intensity profile uniformity, a second correction loop is initiated to recover total beam intensity by adjusting the attenuation filter after the laser source. The complete sensorless correction workflow is illustrated in the flowchart in Fig. S6.

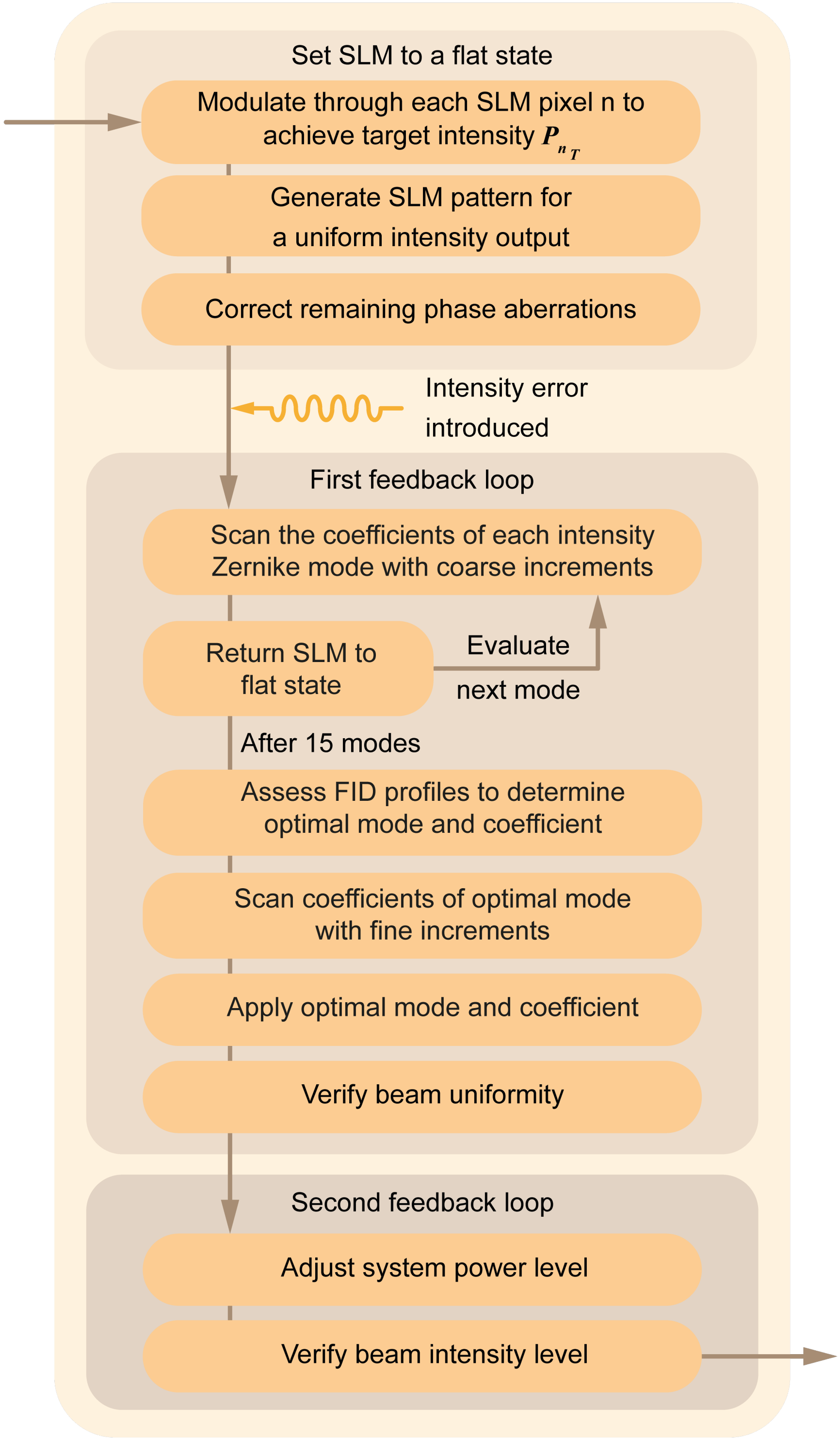

Figure S6: Flowchart illustrating the sensorless correction methodology in the I-AO system. This process includes initial SLM calibration, iterative application and evaluation of intensity Zernike modes, and final adjustments to achieve both intensity uniformity recovery and total intensity restoration.

Supplementary Note 8:

Extended experiment results

Additional experiments were conducted to evaluate the performance of our I-AO methods under real-world conditions. First, we introduced intensity errors using a biomedical sample (fibrotic tissue) placed at a position between the pupil and focal plane to emulate the challenges encountered in practical imaging applications. Second, we demonstrated imaging quality improvements through I-AO in a conventional wide-field microscope using both biomedical (fibrotic tissue) and material (birefringent crystals) samples.

The biomedical sample used in these experiments is a stained fibrotic tissue. The tissue sections are approximately 6 μm thick and were mounted on standard microscopy slides (25 mm × 75 mm × 1 mm). This preparation simulated intensity errors typical of biomedical imaging, showcasing its applicability to complex real-world scenarios.

The material sample consists of birefringent crystals. Following established procedures, the crystal was cleaned, ground into a fine powder, and mounted on standard microscopy slides (25 mm × 75 mm × 1 mm) using a transparent adhesive to ensure stability during the experiments. The resulting sample layer is approximately 10 μm thick. Together, biomedical and material samples facilitated the demonstration of the benefits of I-AO in wide-field microscopy.

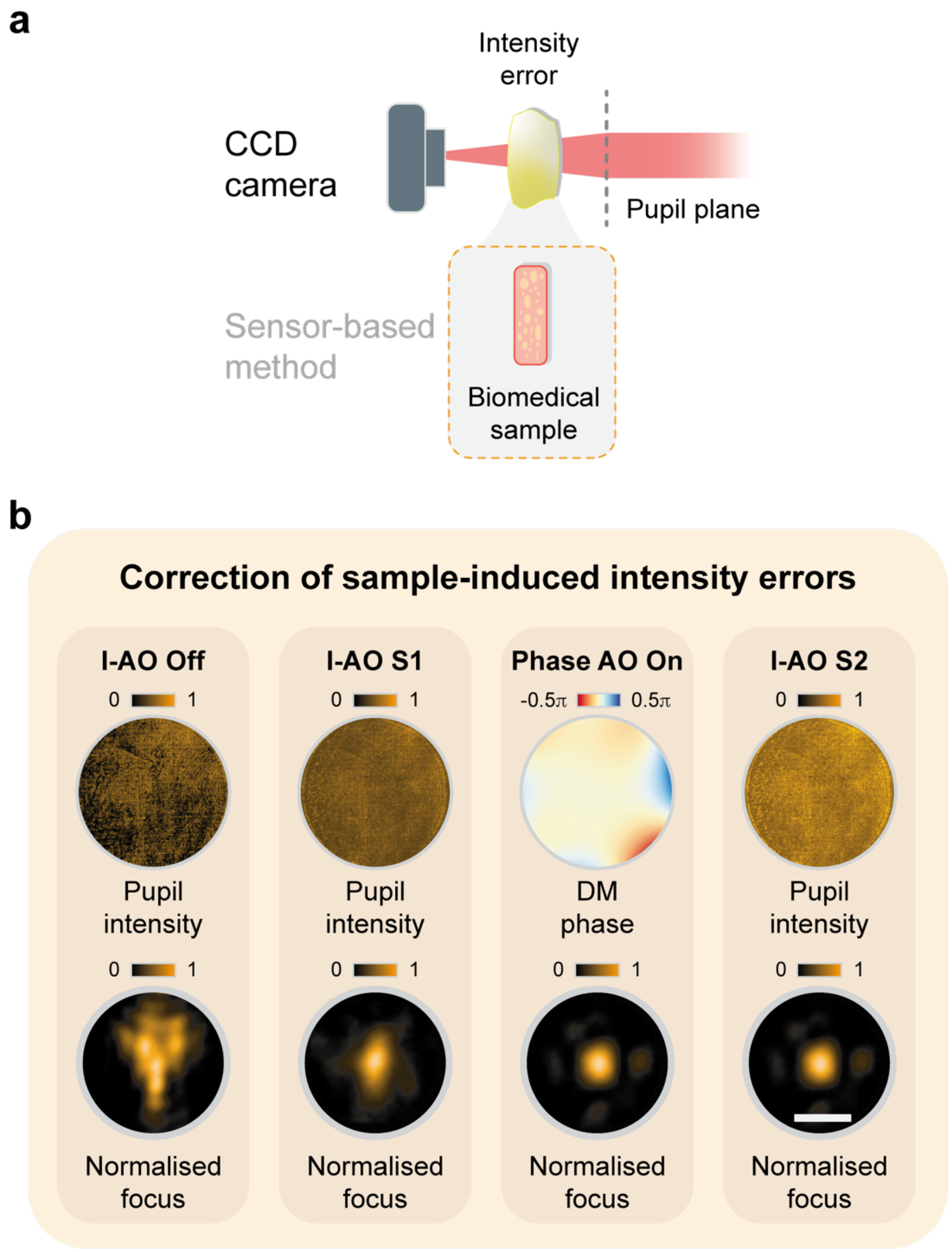

Figure S7: **Correction of intensity errors caused by a biomedical sample using I-AO. (a)** Experiment configuration based on Fig. S1 in **Supplementary Note 1**, demonstrating the correction of intensity errors introduced by a biomedical sample positioned at the sample side (after the pupil plane). **(b)** Performance of the I-AO system in correcting intensity errors introduced by a biomedical sample utilising sensor-based method with a dual feedback loop. The intensity profile of the pupil, the phase applied to the DM, and the profile of the focus for each step are given. The scale bar indicates 30 μm for all normalised focus images.

To evaluate the I-AO performance in practical real-world conditions, intensity errors were introduced by placing a biomedical sample after the pupil plane (Fig. S7(a)). The sensor-based I-AO method was then applied for correction, with results shown in Fig. S7(b). The top row illustrates the pupil intensity distribution before and

after correction, along with the phase pattern on the DM. The bottom row displays the normalised focus at each correction stage.

Without I-AO (I-AO Off), significant intensity non-uniformity is observed at both the pupil and focal plane, primarily due to absorption from the sample. After the first feedback loop (I-AO S1), pupil intensity uniformity is restored, though phase errors from the SLM persist. Conventional sensorless phase AO (Phase AO On) is subsequently applied to correct these residual phase errors, with the associated correction pattern and resulting focal spot shown. The second feedback loop (I-AO S2) compensates for total intensity loss by adjusting the attenuation filter.

Increased beam power is required during the second loop due to sample absorption; however, I-AO remains effective as long as the power stays within system limits. The experiments used an objective lens with NA = 0.1 for the focal plane imaging, where I-AO effectively corrected intensity errors. At a higher NA (approaching ~1; considering dry lens), additional phase and polarisations become unavoidable, which we aim to address by integrating vectorial AO in future work[12].

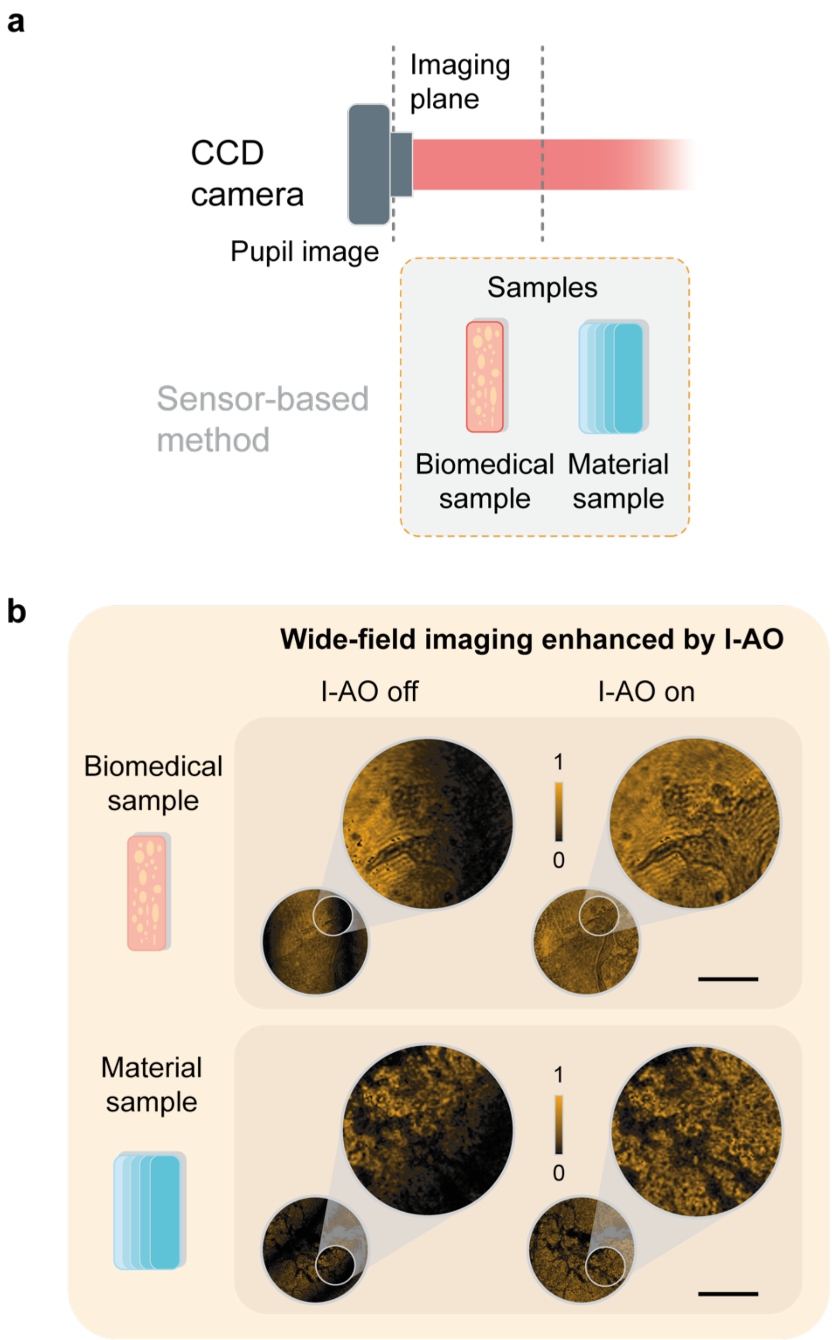

Figure S8: **Improvement in imaging quality with I-AO correction in a wide-field microscope. (a)** The experiment configuration based on Fig. S1 in **Supplementary Note 1** demonstrating the correction of intensity errors introduced by the controllable intensity errors, showing improvements in imaging a biomedical sample and a material sample**. (b)** Comparison of imaging results with (I-AO on) and without (I-AO off) I-AO correction for both samples. The top row shows the results for the biomedical sample, and the bottom row displays the material sample. The left column (I-AO off) reveals non-uniform intensity and reduced contrast, while the right column (I-AO on) demonstrates improved intensity uniformity and enhanced contrast. Magnified insets for each sample highlight the effectiveness of the correction. The scale bar represents 500 μm for the original images and 100 μm for the insets.

To illustrate the benefits of I-AO in a wide-field microscope, further experiments were conducted to enhance the imaging of biomedical and material samples (Fig. S8). Controllable intensity errors were introduced at the location indicated in Fig. S8(a), with samples placed at the conjugate pupil plane after the intensity sensor. The sensor-based I-AO method was then applied for correction. In Fig. S8(b), the top row shows results for the biomedical sample, and the bottom row for the material sample, with magnified insets highlighting correction effectiveness. In both cases, the left column (I-AO Off) shows significant non-uniformities and reduced contrast, while the right column (I-AO On) demonstrates improved image quality with restored illuminations and enhanced contrast.

These experiments demonstrate the effectiveness of I-AO in improving focus spots and overall image quality in real-world conditions, confirming its feasibility for potential practical imaging scenarios requiring precise intensity control.

Reference

[1] Dai, Y., Antonello, J., & Booth, M. J. Calibration of a phase-only spatial light modulator for both phase and retardance modulation. *Optics Express* **27**, 17912–17926 (2019).

[2] Lakshminarayanan, V. & Fleck, A. Zernike polynomials: a guide. *Journal of Modern Optics* **58**, 545–561 (2011).

[3] Chipman, R., Lam, W.S.T., & Young, G. Polarized Light and Optical Systems (CRC Press, 2018).

[4] Theocaris, P. S. & Gdoutos, E. E. Matrix theory of photoelasticity (Springer, 2013).

[5] Goldstein, D. H. Polarized light (CRC Press, 2011).

[6] Steane, A. M. An introduction to spinors. Preprint at: https://doi.org/10.48550/arXiv.1312.3824 (2013).

[7] Carl, M. Influence of polarization aberrations on point images. *Journal of the Optical Society of America* A **34**, 967–974 (2017).

[8] Gutiérrez-Vega, J. C. Pancharatnam–Berry phase of optical systems. *Optics Letters* **36**, 1143–1145 (2011).

[9] He, C., Antonello, J. & Booth, M.J. Vectorial adaptive optics. *eLight* **3**, 23 (2023).

[10] Pancharatnam, S. Generalized theory of interference and its applications. *Proceedings of the Indian Academy of Sciences* **44**, 398–417 (1956).

[11] Debarre, D., Booth, M. J. & Wilson, T. Image based adaptive optics through optimisation of low spatial frequencies. *Optics Express* **15**, 8176-8190 (2007).

[12] He, C., Antonello, J. & Booth, M. J. Vectorial adaptive optics. *eLight* **3**, 23 (2023).
